# Comparative Efficacy of Melatonin and Brassinolide in Mitigating the Adverse Effects of Cadmium on *Wolffia arrhiza*

**DOI:** 10.3390/ijms26020692

**Published:** 2025-01-15

**Authors:** Magdalena Chmur, Andrzej Bajguz

**Affiliations:** Department of Biology and Plant Ecology, Faculty of Biology, University of Bialystok, Ciolkowskiego 1J, 15-245 Bialystok, Poland; m.chmur@uwb.edu.pl

**Keywords:** antioxidants, phytohormones, phytochelatins, primary metabolites, stress markers

## Abstract

Melatonin (MT) and brassinolide (BL) are phytohormones that regulate various physiological processes in plants. This study investigates their effects on *Wolffia arrhiza* when exposed to cadmium (Cd). Plant hormones were quantified using liquid chromatography-mass spectrometry, while photosynthetic pigments and phytochelatins (PCs) were analyzed through high-performance liquid chromatography. Protein, monosaccharide levels, and antioxidant activities were also spectrophotometrically measured. The findings reveal that MT and BL treatment decreased Cd accumulation in *W. arrhiza* compared to plants only exposed to Cd. MT was particularly effective in reversing Cd-induced growth inhibition and reducing stress markers more significantly than BL. It also enhanced antioxidant activity and maintained higher levels of photosynthetic pigments, proteins, and sugars. Although BL was less effective in these aspects, it promoted greater synthesis of glutathione and PCs in Cd-exposed duckweed. Overall, both MT and BL alleviate the negative impact of Cd on *W. arrhiza*, confirming their crucial role in supporting plant health under metal stress conditions.

## 1. Introduction

The growth of the population and, consequently, industrial and agricultural development have led to an increase in environmental contamination by heavy metals; for example, cadmium (Cd) is one of the most damaging trace elements for plants, animals, and humans [1]. In plants, Cd can bind with biologically essential micro- and macroelements, impairing their biological functions. Exposure to Cd reduces or inhibits growth and impairs biochemical processes such as transpiration, cellular respiration, and photosynthesis [2,3]. In addition, poisoning with high doses of Cd leads to the accumulation of reactive oxygen species (ROS), contributing to the formation of malondialdehyde (MDA), which has mutagenic action [4]. In addition to phytohormones, plants possess a variety of defense mechanisms against heavy metal stresses, including the activity of antioxidants [5] and the creation of phytochelatins (PCs) that sequester Cd ions in vacuoles [6,7].

Melatonin (MT) and brassinolide (BL) are phytohormones that regulate many physiological and biochemical processes in plants [8,9,10,11,12,13,14,15]. These phytohormones contribute to cell division and elongation, improve photosynthesis efficiency, and increase the content of proteins, pigments, and monosaccharides in plants [16,17]. Furthermore, MT and BL exhibit antioxidant abilities under abiotic stress conditions, including heavy metals, cold, heat, salinity, and drought [18,19]. These hormones were detected in both algae and vascular plants [12,20,21]. MT is biosynthesized from tryptophan [22,23], while BL is biosynthesized from campestanol (CN) in the CN-dependent pathway or from 22-hydroxy-5α-ergostan-3-one in the CN-independent pathway. BL is also produced in the early C-6 oxidation pathway from CN [23,24].

*Wolffia arrhiza* (L.) Horkel ex Wimm. (Lemnaceae) is the smallest angiosperm, characterized by highly reduced organs and the absence of a root system, stem, and leaves. Flowering occurs rarely, so it usually reproduces vegetatively [25,26]. Its simplified body structure is an excellent adaptation to aquatic life. *W. arrhiza* can absorb heavy metals and other xenobiotics from polluted water [27,28]. Therefore, its ability to accumulate xenobiotics, rapid reproduction, and straightforward cultivation methods make *W. arrhiza* suitable for research and practical use in water purification [29,30]. In this study, we compared the effects of Cd and/or MT or BL on *W. arrhiza*. We hypothesized that: (1) the levels of endogenous phytohormones change when influenced by Cd along with MT or BL; (2) exogenously applied MT and BL can similarly counteract the inhibitory effects of Cd on the growth rate and the concentration of pigments, sugars, and proteins in *W. arrhiza*; (3) both exogenous MT and BL can boost the activity of ascorbate peroxidase (APX), catalase (CAT), glutathione reductase (GR), and superoxide dismutase (SOD), and enhance the levels of glutathione (GSH) and PCs in plants exposed to Cd; (4) treatment with MT or BL leads to reduced H_2_O_2_ and MDA levels in *W. arrhiza* grown with Cd; and (5) the impact of MT and BL on *W. arrhiza* subjected to Cd can vary and depends on the analyzed compounds.

## 2. Results

### 2.1. Melatonin and Brassinolide Increase the Biomass of W. arrhiza Exposed to Cadmium

**Figure 1:**
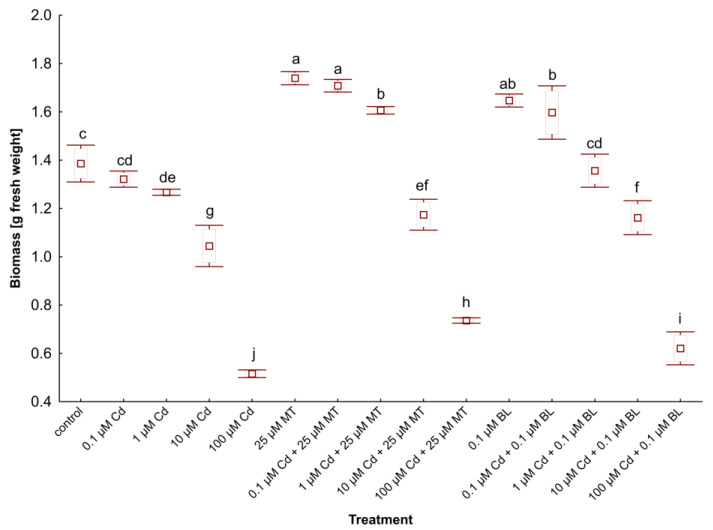
The biomass of *W. arrhiza* treated with Cd or/with MT or BL after 7 days of breeding. The square represents the mean (n = 5, biological replicate). The upper and lower hinges indicate the upper and lower bounds of the 95% confidence interval for the means. The upper and lower whiskers extend from the hinge to the mean ± standard deviation. Means denoted by the same letters are not significantly different (*p* ≥ 0.05), as determined by Tukey’s post hoc test. Abbreviations are explained in the Abbreviations section.

Growth rate is a critical indicator of plant response to heavy metal exposure and phytohormone treatment. This study explored the impact of MT and BL on the growth rate of *W. arrhiza* treated with Cd, as shown in Figure 1.

Initially, each plant variant weighed 1 g. After 7 days of cultivation, the biomass of untreated duckweed rose to 1.37 g. However, applying Cd in concentrations ranging from 0.1 to 100 µM significantly reduced the biomass of *W. arrhiza*, with a notable 68% decrease at the highest Cd concentration (100 µM) compared to the control.

Conversely, the treatment of phytohormones had a beneficial effect on *W. arrhiza* growth. Treatment with 25 µM MT resulted in a 27% increase in plant weight relative to the control, while exposure to 0.1 µM BL led to a 20% increase in duckweed biomass compared to the control. Combining Cd with MT or BL mitigated the metal’s inhibitory effect on duckweed growth. Specifically, 25 µM MT and 0.1 µM BL, alongside varying Cd doses, positively influenced weight compared to duckweed exposed solely to Cd. The biomass of *W. arrhiza* under 25 µM MT supply and either 0.1 or 1 µM Cd was even higher than that of untreated plants. Similarly, combining 0.1 µM BL with 0.1 µM Cd improved growth rates, though the combination of 0.1 µM BL and 1 µM Cd resulted in slightly lower growth than the control. When 100 µM Cd was combined with either 25 µM MT or 0.1 µM BL, biomass increased by 43% and 20%, respectively, compared to duckweed treated only with 100 µM Cd.

### 2.2. Melatonin and Brassinolide Decrease the Cadmium Concentration in W. arrhiza

The influence of MT and BL on the intracellular concentrations of Cd in *W. arrhiza* exposed to Cd is detailed in Table 1.

As expected, the concentration of absorbed Cd ions in duckweed increased in direct proportion to the applied Cd concentrations, with the peak accumulation observed in plants by 100 µM Cd addition. Co-treatment with MT or BL notably reduced the Cd level compared to duckweed exposed to Cd alone. Specifically, administering 25 µM MT or 0.1 µM BL resulted in a 21% and 15% decrease, respectively, in Cd absorption in duckweed by 10 µM Cd supply, in comparison to those grown solely with 10 µM Cd.

Additionally, the analysis of the nutrient medium revealed a decrease in Cd concentration compared to the initial day of exposure. The most significant reduction, a 20% decrease in Cd concentration, was observed in the medium of plants treated with 10 µM Cd. However, when MT or BL was added to the medium, the remaining Cd concentration was higher, indicating a diminished uptake of Cd by the duckweed in the presence of these phytohormones.

### 2.3. Melatonin and Brassinolide Enhance Pigment, Protein, and Monosaccharide Content in W. arrhiza Treated with Cadmium

**Figure 2:**
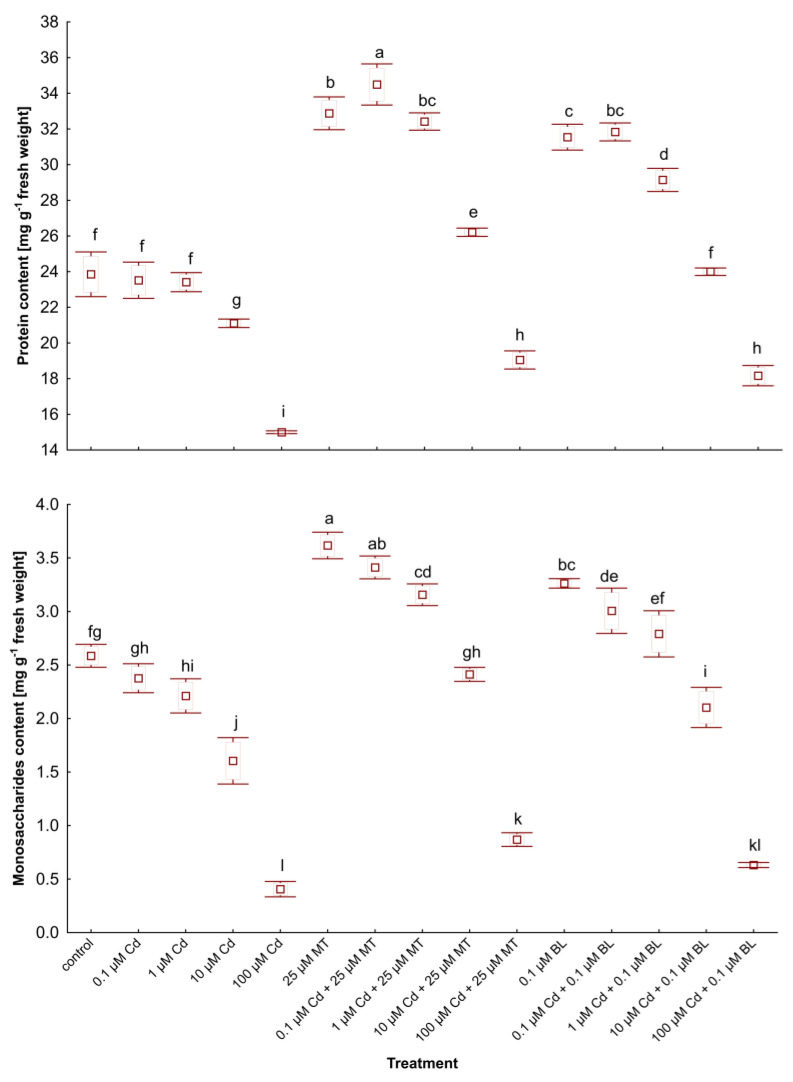
The level of soluble proteins and monosaccharides in *W. arrhiza* treated with Cd or/with MT or BL after 7 days of breeding. The square represents the mean (n = 5, biological replicate). The upper and lower hinges indicate the upper and lower bounds of the 95% confidence interval for the means. The upper and lower whiskers extend from the hinge to the mean ± standard deviation. Means denoted by the same letters are not significantly different (*p* ≥ 0.05), as determined by Tukey’s post hoc test. Abbreviations are explained in the Abbreviations section.

The impact of MT and BL on the levels of photosynthetic pigments, proteins, and monosaccharides in *W. arrhiza* treated with Cd and either MT or BL is detailed in Table 2 and illustrated in Figure 2. Exposure to Cd notably reduced the content of these metabolites, with the most significant decrease observed in plants treated with 100 µM Cd. For example, the chlorophyll *a* level dropped by 60% compared to the control (Table 2). Increasing Cd concentrations caused a significant decline in chlorophyll content, with a marked reduction at 100 µM Cd. α-carotene, β-carotene, neoxanthin, violaxanthin, and lutein levels followed a similar trend, with the highest levels observed in the control plants and a dose-dependent reduction under Cd stress. MT and BL treatments restored carotenoid levels, with 25 µM MT and 0.1 µM BL showing significant protective effects. Zeaxanthin levels decreased less significantly under Cd stress but remained higher in MT- and BL-treated plants, suggesting enhanced photoprotection.

Exposure to BL notably increased the amounts of α-carotene and neoxanthin, while MT was more effective in boosting the synthesis of the remaining compounds (Table 2). Furthermore, the addition of these hormones counteracted the Cd-induced reductions in pigment levels. Specifically, the combination of 25 µM MT with 100 µM Cd resulted in a 46% increase in chlorophylls, compared to plants only exposed to 100 µM Cd.

Protein and monosaccharide contents in duckweed subjected to 100 µM Cd fell by 38% and 85%, respectively, when compared to untreated counterparts (Figure 2). Treatment with 0.1 µM BL and 100 µM Cd enhanced the levels of monosaccharides and proteins by 31%, 57%, and 21%, respectively, versus duckweed treated solely with 100 µM Cd. Comparable trends were observed across other concentrations of Cd when combined with MT or BL. Interestingly, the levels of primary metabolites in *W. arrhiza* with 25 µM MT addition and either 0.1 or 1 µM Cd were even higher than those in untreated plants. Similar results were noted with 0.1 µM BL combined with 0.1 or 1 µM Cd. Treatments with MT and BL mitigated these reductions, with MT showing a more pronounced effect. In plants exposed to Cd and treated with MT, monosaccharide levels significantly exceeded those of the untreated controls at lower Cd concentrations, indicating the phytohormone’s protective role. The results suggest that both MT and BL enhance the plant’s metabolic resilience to Cd stress, with MT having a more robust impact on the recovery of monosaccharide and protein levels.

### 2.4. Melatonin and Brassinolide Mitigate Oxidative Stress in W. arrhiza Exposed to Cadmium

**Figure 3:**
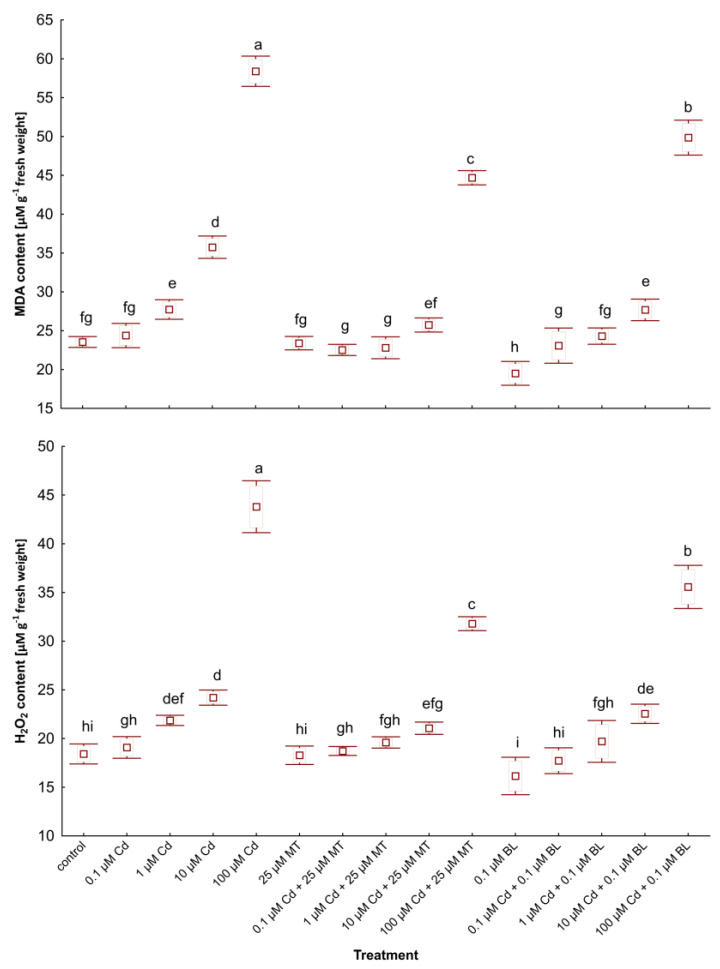
The levels of MDA and H_2_O_2_ in *W. arrhiza* treated with Cd or/with MT or BL after 7 days of breeding. The square represents the mean (n = 5, biological replicate). The upper and lower hinges indicate the upper and lower bounds of the 95% confidence interval for the means. The upper and lower whiskers extend from the hinge to the mean ± standard deviation. Means denoted by the same letters are not significantly different (*p* ≥ 0.05), as determined by Tukey’s post hoc test. Abbreviations are explained in the Abbreviations section.

Oxidative stress, indicated by increased lipid peroxidation and H_2_O_2_ accumulation, is a critical concern for plants exposed to heavy metals. The influence of MT and BL on oxidative stress markers in *W. arrhiza* treated with Cd is depicted in Figure 3. Exposure to Cd significantly elevated the levels of MDA and H_2_O_2_, with the highest increases observed in plants subjected to 100 µM Cd.

Interestingly, treatment with MT alone did not alter stress marker levels compared to the control, whereas adding 0.1 µM BL slightly reduced MDA and H_2_O_2_ contents. The concurrent application of MT or BL with Cd notably decreased the accumulation of these stress markers relative to plants under Cd supply. Specifically, a combination of 100 µM Cd and 25 µM MT resulted in 28% and 24% decreases in H_2_O_2_ and MDA levels, respectively, compared to plants exposed solely to 100 µM Cd. Similarly, duckweed treated with a mix of 100 µM Cd and 0.1 µM BL showed 19% and 15% reductions in H_2_O_2_ and MDA contents, respectively, versus the group without hormone treatment.

Furthermore, duckweed grown with low-dose Cd (0.1 µM) in combination with either hormone displayed lower stress marker levels than the control group despite an increase in these compounds in plants by 0.1 µM Cd addition. However, the scenario differed slightly at 1 µM Cd: MDA accumulation decreased in plants treated with 1 µM Cd and 25 µM MT compared to the control, while the MDA level in plants exposed to 1 µM Cd and 0.1 µM BL was higher than in untreated duckweed.

### 2.5. Melatonin and Brassinolide Enhance the Content of Glutathione and Phytochelatins in W. arrhiza Treated with Cadmium

**Figure 4:**
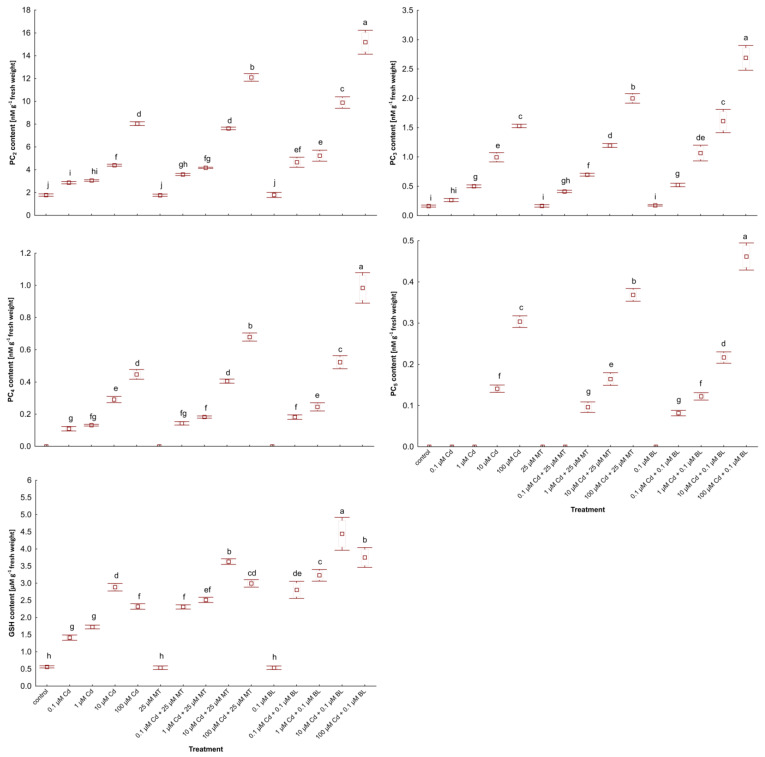
The level of PC_2_, PC_3_, PC_4_, PC_5_, and GSH in *W. arrhiza* treated with Cd or/with MT or BL after 7 days of breeding. The square represents the mean (n = 5, biological replicate). The upper and lower hinges indicate the upper and lower bounds of the 95% confidence interval for the means. The upper and lower whiskers extend from the hinge to the mean ± standard deviation. Means denoted by the same letters are not significantly different (*p* ≥ 0.05), as determined by Tukey’s post hoc test. Abbreviations are explained in the Abbreviations section.

Glutathione (GSH) and phytochelatins (PCs) play crucial roles in detoxifying heavy metals [31,32]. Figure 4 illustrates the levels of GSH and PC_2-5_ in *W. arrhiza* subjected to Cd stress, with or without treatment by MT or BL.

In the control group, GSH and trace amounts of PC_2-3_ were present. Cd exposure markedly increased PCs synthesis, peaking in plants by 100 µM Cd supply. Additionally, GSH levels were elevated in duckweed exposed to 100 µM Cd compared to those treated with lower Cd concentrations (10 µM). Among the PCs, PC_2_ was predominant, while PC_3-5_ was less abundant.

Treatment with 25 µM MT or 0.1 µM BL alone had no significant impact on the synthesis of these thiol compounds. However, co-treatment with Cd and either hormone notably enhanced PCs content. BL was more effective than MT in stimulating the synthesis of thiol compounds in Cd-stressed plants. Specifically, in duckweed treated with 0.1 µM BL under 100 µM Cd addition, the levels of GSH and PC_2-5_ increased by 63%, 89%, 77%, 122%, and 53%, respectively, compared to plants subjected to 100 µM Cd alone. Meanwhile, the inclusion of 25 µM MT led to increases of 28%, 50%, 30%, 54%, and 20% in GSH and PC_2-5_ levels, respectively, under the same Cd stress conditions.

Interestingly, introducing 1 µM Cd with either 0.1 µM BL or 25 µM MT triggered the synthesis of PC_5_. The total PCs level reached 19 nM/g in duckweed treated with 100 µM Cd and 0.1 µM BL and 15 nM/g in those under 100 µM Cd and 25 µM MT supply.

### 2.6. Melatonin and Brassinolide Promote Antioxidant Activities in W. arrhiza Under Cadmium Stress

**Figure 5:**
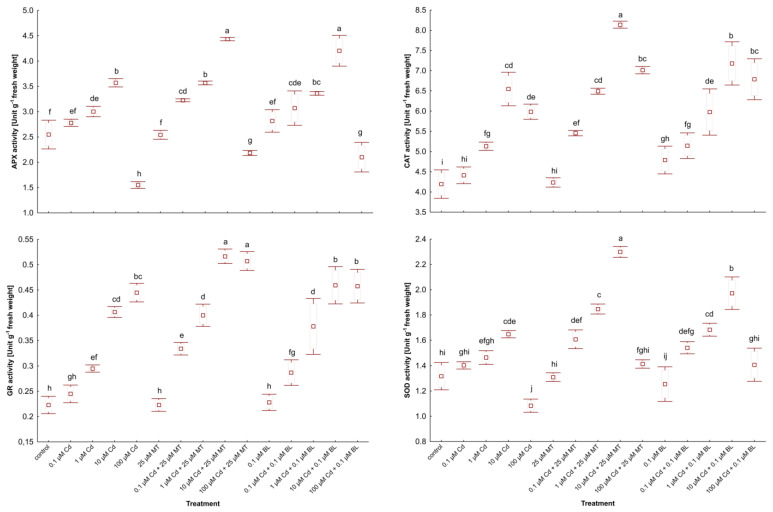
The activity of APX, CAT, GR, and SOD in *W. arrhiza* treated with Cd or/with MT or BL after 7 days of breeding. The square represents the mean (n = 5, biological replicate). The upper and lower hinges indicate the upper and lower bounds of the 95% confidence interval for the means. The upper and lower whiskers extend from the hinge to the mean ± standard deviation. Means denoted by the same letters are not significantly different (*p* ≥ 0.05), as determined by Tukey’s post hoc test. Abbreviations are explained in the Abbreviations section.

The capacity to synthesize antioxidants is crucial for plant responses to different environmental stresses [33,34]. The impact of MT and BL on ascorbate peroxidase (APX), catalase (CAT), glutathione reductase (GR), and superoxide dismutase (SOD) in *W. arrhiza* treated with Cd is illustrated in Figure 5.

Exposure to low levels of Cd (0.1 µM) modestly increased antioxidant activity relative to unstressed duckweed. However, exposure to 10 µM Cd significantly elevated the activities of these enzymes, except for GR, which reached its peak activity under 100 µM Cd stress.

Treatment with MT alone did not significantly alter antioxidant activity, while exposure to BL alone slightly increased the activities of APX and CAT. Conversely, co-treatment with 25 µM MT or 0.1 µM BL and Cd markedly enhanced the activities of these enzymes. Overall, MT application resulted in a more pronounced stimulation of antioxidant synthesis in Cd-stressed plants than BL. Specifically, GR activity in duckweed treated with 10 µM Cd and either 25 µM MT or 0.1 µM BL was elevated by 27.5% and 15%, respectively, compared to plants without hormone treatment. Additionally, SOD activity in plants under 10 µM Cd and 25 µM MT addition was 15% higher than in those under 10 µM Cd and 0.1 µM BL supply.

### 2.7. Melatonin and Brassinolide Affect Phytohormone Levels in W. arrhiza Treated with Cadmium

**Figure 6:**
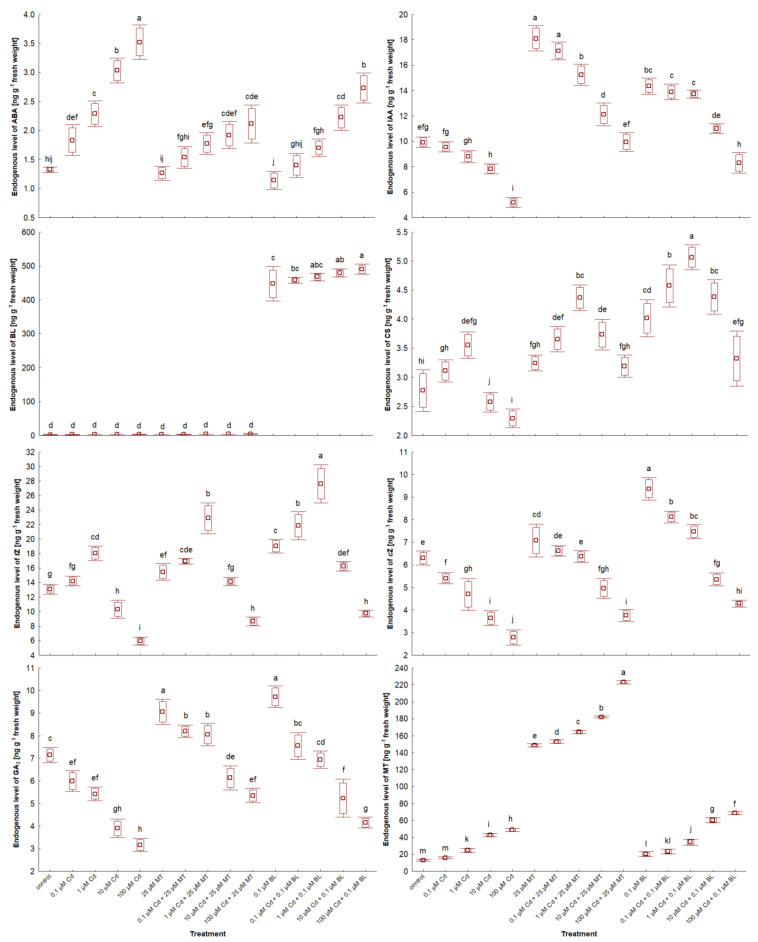
The level of selected free phytohormones in *W. arrhiza* treated with Cd or/with MT or BL after 7 days of breeding. The square represents the mean (n = 5, biological replicate). The upper and lower hinges indicate the upper and lower bounds of the 95% confidence interval for the means. The upper and lower whiskers extend from the hinge to the mean ± standard deviation. Means denoted by the same letters are not significantly different (*p* ≥ 0.05), as determined by Tukey’s post hoc test. Abbreviations are explained in the Abbreviations section.

**Figure 7:**
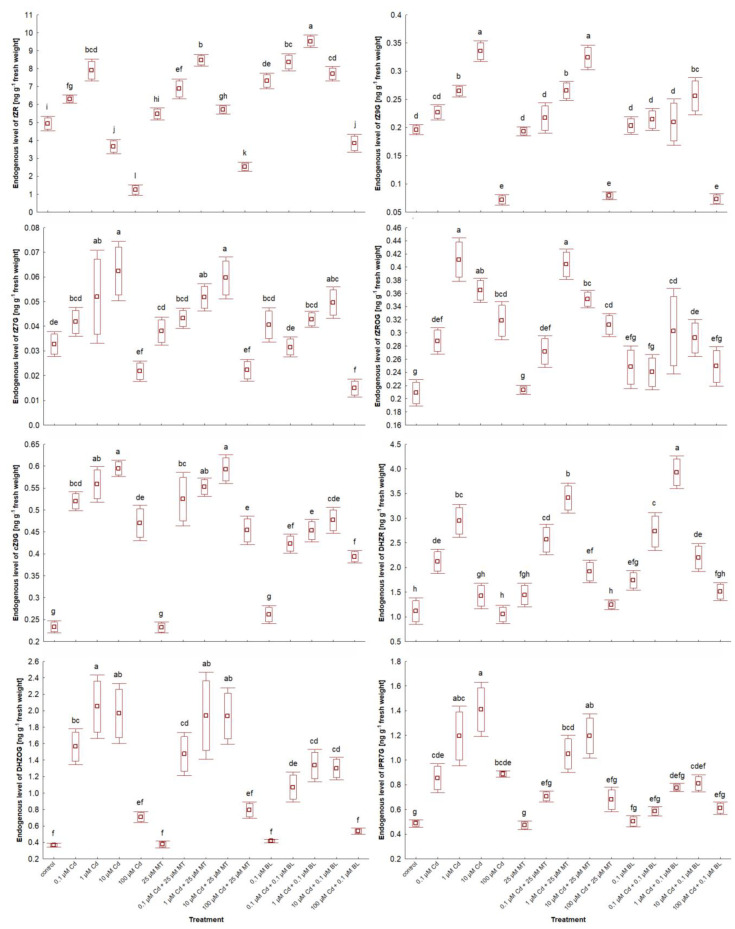
The level of selected conjugated cytokinins in *W. arrhiza* treated with Cd or/with MT or BL after 7 days of breeding. The square represents the mean (n = 5, biological replicate). The upper and lower hinges indicate the upper and lower bounds of the 95% confidence interval for the means. The upper and lower whiskers extend from the hinge to the mean ± standard deviation. Means denoted by the same letters are not significantly different (*p* ≥ 0.05), as determined by Tukey’s post hoc test. Abbreviations are explained in the Abbreviations section.

The impact of MT and BL on the endogenous levels of phytohormones in *W. arrhiza* exposed to Cd is depicted in Figure 6 and Figure 7 and Table 3. LC-MS/MS analysis identified a spectrum of phytohormones, including ABA; two auxins (AXs), namely IAA and PAA; nine brassinosteroids (BRs), including BL, EBL, HBL, norBL, CT, CS, ECS, TY, and 6dTY; seven free bases of cytokinins (CKs), such as tZ, cZ, DHZ, iP, iPR, oT, and mT; eight CK conjugates, including tZR, tZ9G, tZ7G, tZROG, cZ9G, DHZR, DHZOG, and iPR7G; along with GA_3_ and MT, totaling twenty-nine identified phytohormones.

In the untreated plants, the highest concentrations were observed for tZ (13.082 ng/g), MT (12.934 ng/g), and IAA (9.943 ng/g), whereas the lowest were for mT (0.015 ng/g), ECS (0.028 ng/g), and 6dTY (0.032 ng/g).

The response to Cd ranged from 0.1 µM to 100 µM, depending on the phytohormone. All doses of Cd stimulated the levels of ABA, most BRs, and glucoside CK conjugates, except for tZ79 and tZ9G in duckweed exposed to 100 µM Cd. This effect was also observed for aromatic CK-free bases and MT. For example, 100 µM Cd exposure resulted in nearly a 4-fold increase in MT levels and about a 2.5-fold increase in ABA levels compared to untreated plants, with the concentration of these phytohormones generally rising with increasing Cd levels. Conversely, 0.1 µM–100 µM Cd treatment significantly reduced the levels of AXs, cZ, norBL, ECS, and GA_3_. Specifically, 100 µM Cd exposure led to a 52% decrease in IAA levels and a 44% reduction in GA_3_ levels. The effects of Cd on other phytohormones varied in a concentration-dependent manner, with 0.1 and 1 µM Cd significantly increasing tZ and tZR levels, while 10 and 100 µM Cd exposures notably reduced their levels, a pattern also seen for DHZ and iP.

Exposure to 25 µM MT or 0.1 µM BL alone either stimulated or neutralized phytohormone levels. MT treatment led to an 11-fold increase in endogenous MT content due to absorption by *W. arrhiza*. It also doubled the level of IAA and increased GA_3_ content by 22% compared to the control. MT had a minor stimulatory effect on the level of BRs, isoprenoid-free bases, and ribosides of CKs but did not significantly affect the remaining CKs and ABA. Conversely, BL treatment increased all detected phytohormones except for ABA and aromatic-free CK bases. For instance, the endogenous MT levels rose by 65% in plants with a 0.1 µM BL supply. The combined treatment with Cd and either MT or BL increased the levels of AXs, BRs, most CK-free bases, GA_3_, and MT but reduced ABA levels compared to the Cd treatment alone. CK glucoside levels decreased significantly after the combined application of Cd and BL compared to after Cd alone or the combination of Cd with MT. In contrast, the levels of free bases and ribosides of CKs and BRs were enhanced with the combined treatment of Cd and BL compared to Cd alone and the mixture of Cd with MT. Meanwhile, the most pronounced effects on IAA, GA_3_, and MT levels were observed in plants under Cd and MT addition.

## 3. Discussion

Plants are becoming increasingly exposed to heavy metal pollutants. These elements are released into aquatic environments primarily due to anthropogenic activities [35]. Cd poisoning inhibits plant growth and cell division, reduces metabolite content, and contributes to increased stress markers [3]. *W. arrhiza*, an aquatic plant devoid of roots, can remarkably biosorb chemical compounds from water habitats [27]. In this study, significant Cd absorption from the medium was observed. The intracellular content of Cd in *W. arrhiza* increased in proportion to the dose of Cd added to the medium. Moreover, the application of both 25 µM MT and 0.1 µM BL resulted in reduced Cd concentration in the plant, which could be attributed to accelerated PC synthesis. Compared to BL, a slightly more significant decrease in Cd absorption was observed in duckweed treated with MT. Previous research has shown that MT can reduce Cd concentration by influencing the expression of genes responsible for transport, consequently limiting the entry of Cd ions into plant cells [36]. Another study suggested that the decreased Cd uptake could be due to MT’s ability to modulate ABC transporter activity, acting as a Cd extrusion pump [37]. Reduced Cd accumulation in response to 25 and 50 µM MT treatment was also observed in the roots, stems, and leaves of *Pennisetum glaucum* [38], as well as in seedlings of *Oryza sativa* [39]. Conversely, exposure to BL resulted in a decrease in intracellular Cd content in the shoots of *Nasturtium officinale* [40], and exogenous EBL reduced Cd accumulation in the roots and leaves of Kentucky bluegrass (*Poa pratensis*) [41].

The accumulation of Cd directly affects the growth and metabolism of plants [3]. The first visible effect of Cd exposure is the inhibition of cell division and a decrease in *W. arrhiza* biomass. In this study, treatment with Cd led to reduced plant growth, and exposure to 100 µM Cd resulted in the death of most *W. arrhiza* cultures. In addition to growth inhibition, a decrease in metabolites, such as photosynthetic pigments, proteins, and monosaccharides, was also observed in response to Cd treatment. Chlorophylls, essential green pigments that mainly absorb light for ATP synthesis, predominantly include chlorophyll *a* and *b*. Environmental conditions and phytohormones influence the synthesis and degradation of chlorophylls. Measuring their content can determine the rate of photosynthesis in plants. Carotenoids, which possess antioxidant activity, protect chlorophylls against photo-oxidative damage [13] and play a crucial role in the light phase of the photosynthesis process [42]. Exposure to Cd resulted in a reduced level of these pigments in *W. arrhiza*. Cd can bind with photosynthetic pigment elements, such as Ca^2+^, Mg^2+^, K^+^, and Fe^2+^, potentially causing chlorosis and chlorophyll degradation [43]. Additionally, exposure to Cd leads to elevated ROS synthesis, which contributes to the peroxidation of chloroplast membranes [44]. Violaxanthin, astaxanthin, zeaxanthin, and lutein are key carotenoids analyzed in this study, highlighting their critical roles in the physiological response of *W. arrhiza* to Cd stress. Violaxanthin, as part of the xanthophyll cycle, plays a significant role in photoprotection by dissipating excess absorbed light energy as heat, thereby preventing photoinhibition and oxidative damage to the photosynthetic apparatus. The conversion of violaxanthin to zeaxanthin under stress conditions enhances this protective mechanism, as observed in our results, which emphasize the importance of the xanthophyll cycle in mitigating Cd-induced oxidative stress [45,46]. Zeaxanthin, in addition to its photoprotective function, stabilizes the photosynthetic machinery, ensuring sustained photosynthetic efficiency even under metal stress [46]. Our data reveal an increase in zeaxanthin levels when plants were treated with MT or BL, suggesting that these phytohormones further enhance photoprotection mechanisms. Astaxanthin, although less common in higher plants, exhibits potent antioxidant properties that protect chloroplast membranes from lipid peroxidation induced by ROS. Studies have demonstrated that astaxanthin can inhibit lipid peroxidation by neutralizing ROS, thereby protecting cellular components from oxidative damage. This protective effect is particularly significant in environments where plants are exposed to abiotic stresses that elevate ROS levels [47]. Our findings demonstrate a correlation between elevated astaxanthin levels and improved oxidative stress tolerance in *W. arrhiza*, particularly in plants exposed to Cd and treated with phytohormones. Lutein, one of the most abundant carotenoids in plants, is essential for light harvesting and photoprotection [46]. In this study, lutein levels were notably higher in plants treated with MT or BL under Cd stress compared to untreated plants, underscoring its role in maintaining photosynthetic pigment stability and scavenging ROS. These results highlight the importance of carotenoids in the stress response of *W. arrhiza*, particularly their involvement in photoprotection, ROS scavenging, and maintaining photosynthetic efficiency. The observed modulation of violaxanthin, astaxanthin, zeaxanthin, and lutein by MT and BL treatments underscores their potential as protective agents against Cd toxicity in aquatic plants. These carotenoids not only contribute to protecting photosynthetic efficiency and mitigating oxidative damage but also enhance overall plant stress tolerance. Their roles in photoprotection and antioxidant defense are particularly vital for plants like *W. arrhiza* under abiotic stresses such as Cd toxicity.

Monosaccharides are an essential energy and carbon reservoir necessary for all biochemical processes in plants. During photosynthesis, monosaccharides (mainly glucose) are synthesized. Thus, a decrease in sugar levels is associated with the degradation of photosynthetic pigments [48]. Protein degradation in *W. arrhiza* is directly related to Cd action and DNA damage caused by Cd ions [49]. However, the application of MT and BL mitigated the toxic effect of Cd on the content of primary metabolites in *W. arrhiza*. MT significantly downregulates the expression of enzymes responsible for chlorophyll degradation, such as chlorophyllase and pheophytinase [50]. Meanwhile, BL activates enzymes involved in chlorophyll biosynthesis and enhances light-capturing efficiency [13]. In our research, the level of primary metabolites, except for α-carotene, neoxanthin, and cryptoxanthin, was more efficiently enhanced in response to MT than BL treatment. These findings are consistent with the reports of Wang et al. [51], Sami et al. [52], Tousi et al. [53], and Ahmed et al. [54].

Exposure to Cd leads to an increase in the synthesis of ROS, including H_2_O_2_, resulting in intensified lipid peroxidation and elevated production of MDA in *W. arrhiza*. Elevated ROS levels enhance the activity of antioxidant enzymes responsible for scavenging ROS. APX catalyzes the conversion of H_2_O_2_ to H_2_O using ascorbate as a substrate, while SOD transforms the O_2_ radical into H_2_O_2_, which is then broken down into H_2_O and O_2_ by CAT [51]. Meanwhile, GR facilitates the reduction of GSSG to GSH, playing a role in the detoxification process of heavy metals [1]. Our study revealed that the levels of stress markers and the activities of antioxidant enzymes significantly increased in *W. arrhiza* in response to Cd treatment compared to the control. The heightened activities of APX and CAT are associated with increased H_2_O_2_ levels in plants exposed to Cd.

MT and BL positively impact ROS scavenging and antioxidant capacity. This study found that the application of these phytohormones reduced the levels of H_2_O_2_ and MDA in *W. arrhiza* treated with Cd, compared to plants exposed only to this heavy metal. Furthermore, treatment with MT or BL boosted antioxidant enzyme activities in response to Cd exposure, with a more pronounced antioxidant action observed for MT than BL. These findings align with those of Khan et al. [55], who reported a reduction in stress markers and an increase in antioxidant activity in Cd-treated cotton seedlings after the addition of 15 µM MT. Similarly, other research [56] showed decreased stress marker content and increased antioxidant activity in Cd-treated *Brassica juncea* seedlings following EBL supplementation. Our analysis highlights the protective roles of BL and MT against oxidative stress and lipid peroxidation.

PCs are crucial thiol compounds that detoxify heavy metals in plants. Their function involves chelating metal ions in the plant cytosol and sequestering them into the vacuole as high molecular weight complexes, making the metals less toxic. The presence of cadmium (Cd) ions in the plant cytosol is a major inducer of PCs synthesis. The most abundant and stable PC form, PC_2_, is produced by linking a γ-glutamylcysteine (γ-Glu-Cys) molecule with GSH. PC_3-5_ are synthesized by connecting γ-Glu-Cys with another PC acceptor molecule [6,57]. Heavy metals, including Cd, can increase the synthesis of PCs through the induction of genes that encode PC synthase, a key enzyme in the biosynthesis of PCs [1]. Our study revealed that Cd exposure significantly stimulated the biosynthesis of PC_2-5_ in *W. arrhiza*. However, the GSH content was lower in plants treated with 100 µM Cd than those exposed to 10 µM Cd, which was attributed to the utilization of GSH for PCs synthesis. Similar results were reported for the moss *Leptodictyum riparium* [58]. Furthermore, our findings demonstrated that both MT and BL increased the biosynthesis of PCs in plants under Cd stress, with BL having a more pronounced effect than MT in intensifying the synthesis of PCs in response to Cd exposure. Amjadi et al. [1] found that PCs content in Cd-treated seedlings of *Carthamus tinctorius* increased following MT addition, while Talarek-Karwel et al. [59] observed a stimulative effect of EBL on PCs levels in the green alga *Acutodesmus obliquus* exposed to lead (Pb). In summary, the enhanced synthesis of PCs and the accumulation of Cd-PC complexes, stimulated by MT and BL, could be essential mechanisms for Cd tolerance in plants [60].

The interaction between MT or BL and other phytohormones under Cd stress conditions is not well understood, especially on the endogenous phytohormone levels. Generally, both MT [61] and BL [62] can interact with other phytohormones, enhancing plant tolerance to stress. Therefore, the primary focus of this work is to compare the effects of MT and BL on the content of phytohormones in *W. arrhiza* treated with Cd. It was reported that ABA, two AXs, i.e., IAA and PAA; nine BRs, including BL, EBL, HBL, norBL, CT, CS, ECS, TY, and 6TY; fifteen CKs, e.g., tZ, and tZR; GA_3_ and MT were detected in *W. arrhiza*.

In plants subjected to biotic and abiotic stresses, the biosynthesis of ABA, a stress-signaling phytohormone, increases [63]. For instance, the endogenous level of ABA rose in *Kosteletzkya pentacarpos* exposed to 10 µM Cd [64], and in the macroalga *Gracilariopsis lemaneiformis* treated with 100 µM Cd [65]. ABA has been found to interact antagonistically with MT and BL [66]. In our study, Cd treatment significantly increased the content of ABA, while adding phytohormones reduced ABA levels in *W. arrhiza* treated with Cd. BL caused a more significant decline in ABA content than MT. This is corroborated by existing research, which illustrates that exogenous application of MT reduced ABA content in grains of summer maize under water deficit conditions [9], and in perennial ryegrass under heat stress [67]. Similarly, exogenous BL decreased ABA accumulation in the leaves of *Arabidopsis thaliana* [68]. ABA accumulation was positively correlated with ROS synthesis. For example, a decreased ABA level was associated with reduced H_2_O_2_ accumulation [69]. This relationship was confirmed in our study, where exogenous MT and BL reduced both ABA and stress marker concentrations in *W. arrhiza* exposed to Cd.

AXs are essential, multifunctional phytohormones that regulate plant growth and respond to environmental stresses [70]. Interestingly, both MT and IAA were synthesized from the amino acid tryptophan. Recent research has confirmed that Cd significantly reduces the concentration of AXs in *W. arrhiza*. Similarly, exposure to 50 µM Cd significantly declined AXs content in *Populus canescens* [71]. Zhou et al. [64] also reported a decrease in AXs content in *Kosteletzkya pentacarpos* treated with Cd. However, an opposite effect was observed in *W. arrhiza* treated with phytohormones. Both BRs and MT act synergistically with AXs. Phosphorylation mediated by BIN2 (brassinosteroid insensitive 2) protein kinase stimulates the expression of BR-regulated genes that promote AXs synthesis. The IAA/AXs genes also involve BR-regulated AXs synthesis [62]. Genetic associations between MT and AXs have been described [72]. The endogenous levels of IAA and PAA significantly increased in *W. arrhiza* with the addition of MT or BL. Similarly, exogenous MT induces AXs biosynthesis in the hypocotyl of tomato seedlings [73], and the IAA content was also higher in maize grain treated with MT [9]. The inhibitory effect of Cd on AXs synthesis was reversed through the application of phytohormones, with a more pronounced effect observed for MT than BL. Niu et al. [41] reported the addition of EBL to Cd-treated seedlings of Kentucky bluegrass caused an increase in IAA content compared to seedlings grown with Cd alone.

Cd stress significantly increased the levels of MT and moderately elevated the content of BRs in *W. arrhiza*. This is consistent with previous research demonstrating the stimulating effect of Cd on MT synthesis in rice seedlings [74]. Our study further explored how exogenous application of MT influences the endogenous levels of BRs and how exogenous BL affects the endogenous content of MT, revealing a positive correlation between these phytohormones. These findings align with the work of Hwang and Back [75], who found that exogenous BL induced the biosynthesis of MT in rice seedlings under both Cd stress and non-stress conditions. Additionally, exogenous MT was shown to induce the expression of several BR biosynthetic genes, while the content of bioactive BRs was reduced in melatonin-deficient transgenic rice plants [76]. Lee and Back [77] also reported a significant decrease in bioactive BRs in melatonin-deficient rice plants, underscoring the positive association between MT and BR levels. The stimulating effect of 0.1 µM EBL on the BRs content in *Hordeum vulgare* seedlings further demonstrates this association [78]. These findings suggest a complex interplay between MT and BRs, highlighting their synergistic roles in enhancing plant resilience to Cd stress.

Naturally occurring CKs derive from the adenine purine base and possess either an aromatic or isoprenoid side chain at the *N*^6^ position. The major forms of CKs are isoprenoid-free bases, such as tZ and cZ, which are the most biologically active types of CKs. The most abundant CKs with an aromatic side chain are topolins. CKs often form conjugates with glucose and/or ribose attached to the purine ring [79]. The *cis* configurations of isoprenoid CKs are most commonly synthesized through the degradation of tRNA [80]. The primary role of CKs in plants includes inducing cell division and preventing cell aging [81]. Additionally, glucosides are a storage form, providing a constant supply of free CK bases [82]. In our studies, the effect of Cd on the endogenous level of CKs was concentration-dependent. Generally, treatment with 10 and 100 µM Cd decreased the content of free CK bases below levels detected in the control group. However, the level of glucose conjugates under Cd stress was higher compared to the control. The rate of glucoside conjugates increases under exposure to high levels of heavy metals [83,84]. According to Hashem [85], exposure to 100 µM Cd significantly reduced the content of tZ and tZR in soybean (*Glycine max*), and Cd stress also decreased the concentration of tZR in the leaves of wheat [86]. Furthermore, the content of free CK bases was reduced, while the level of CK-*O*-glucosides increased in *Kosteletzkya pentacarpos* exposed to 10 µM Cd [64]. Our results also indicated that exogenous MT and BL increased the content of the free bases and ribosides of CKs in both unexposed and Cd-exposed groups of plants, with a stronger effect observed for BL than MT. Research by Kudryakova et al. [87] showed that applying BL increased CK content in *Arabidopsis thaliana*, likely by inducing the expression of CK biosynthesis genes. Similarly, a high concentration of 300 µM Cd also caused a decrease in tZR and iP levels in Kentucky bluegrass, which was mitigated after the addition of EBL [41]. Other studies have demonstrated elevated CK levels in wheat seedlings treated with EBL [88]. Exogenous treatment with 20 µM MT increased the accumulation of tZ in rice seedlings and upregulated the expression of CK biosynthetic genes [89]. Zhang et al. [67] reported that the content of iP and tZR in *Lolium perenne* exposed to heat stress increased after adding MT. The amount of tZ and tZR also rose after treatment with MT in maize grains growing under semiarid conditions [9].

GA_3_ is a phytohormone belonging to the group of GAs, known for its biological activity. The chemical structure of GA_3_ features a carboxylic acid skeleton with a carboxyl group at the C-6 position and a hydroxyl group at the C-3 position [90]. The endogenous level of GA_3_ significantly decreased in *W. arrhiza* exposed to Cd, consistent with the analyses of Guo et al. [86] and Zhou et al. [64]. Conversely, an opposite effect was observed in plants exposed to phytohormones. MT or BL, in conjunction with GAs, interact genetically at the level of proteins and DNA. Treatment with MT and BL upregulates the expression of GA20ox genes involved in GA_3_ synthesis [62,91]. In our studies, MT was more effective than BL at mitigating the reduction of GA_3_ levels. The content of this phytohormone was also reduced in *Brassica napus* seedlings under salt stress conditions but was partially restored after treatment with MT [91]. Other studies have shown that the endogenous level of GA_3_ increased in *Leymus chinensis* after the application of BL [92].

Determining which phytohormone, MT or BL, is better suited depends on how effectiveness is defined. If effectiveness is evaluated based on the ability to stimulate plant growth and metabolism at the lowest concentration, BL could be considered more efficient, as it achieves notable effects at lower concentrations than MT. Conversely, if the criterion is the broad range of benefits induced, regardless of dosage, MT might be viewed as offering a more comprehensive protective role against stressors like Cd. At relatively higher concentrations, MT provides stronger protection and stimulates various aspects of plant physiology to a greater extent than BL, which may make it preferable in specific experimental or application scenarios. Thus, the assessment of effectiveness depends on the intended goal—whether to achieve effects with minimal concentration or to maximize the plant’s overall positive response, irrespective of dosage. In practical terms, MT and BL each have unique advantages and can be applied complementarily, depending on the specific needs and conditions. This study confirms that MT exhibits a greater capacity to mitigate the adverse effects of Cd on the growth, photosynthetic pigments, proteins, sugars, and stress markers of *W. arrhiza* compared to BL. However, BL demonstrates a stronger ability to enhance the synthesis of detoxifying compounds like GSH and PCs. The broader impact of MT on growth, metabolism, and stress marker reduction highlights its pronounced beneficial effects under Cd stress. The reduction of Cd toxicity by both MT and BL was observed in *W. arrhiza* after 7 days, but future long-term studies are needed to evaluate the sustained effectiveness and potential cumulative benefits of these phytohormones over extended periods [4,30,93].

The difference in optimal concentrations for MT (25 µM) and BL (0.1 µM) underscores disparities in their potency and efficacy. BL’s ability to achieve its effects at significantly lower concentrations implies higher potency per unit mass, offering potential advantages in cost and application efficiency, particularly in scenarios requiring minimal input. On the other hand, MT’s more comprehensive protective effects at higher concentrations suggest its suitability in situations where maximal stress alleviation is required, even at the expense of higher dosages. Accordingly, the interpretation that MT has a stronger overall impact on the growth and metabolism of *W. arrhiza* exposed to Cd is valid, with the understanding that efficiency depends on the context. If efficiency is measured by the breadth of beneficial effects, MT is superior. However, if it is defined by the impact per unit concentration, BL’s efficacy at lower concentrations could be deemed more efficient. This nuanced view supports the conclusion that both phytohormones possess distinct strengths and potential applications, which can complement each other depending on environmental conditions and specific objectives [4,30,93].

## 4. Materials and Methods

### 4.1. Plant Growth Conditions

*W. arrhiza* was grown in a sterile glass vessel containing 200 mL of Hutner [94] medium for 7 days under stable conditions: a 16 h photoperiod (photon flux of 100 µmol/m/s), 22.0 ± 0.5 °C, and 65% humidity. Throughout the experiment, duckweed cultures were treated with 0.1, 1, 10, and 100 µM Cd; 25 µM MT; 0.1 µM BL; and mixtures of all Cd concentrations with 25 µM MT or 0.1 µM BL. A control group of untreated plants was also included. Concentrations of 25 µM MT and 0.1 µM BL were selected based on previous research by Chmur and Bajguz [4,93] as optimal for the growth and compound content analysis in *W. arrhiza*. The preliminary metal solution was prepared by dissolving the CdCl_2_ powder in distilled water, while BL and MT were dissolved in 70% (*v*/*v*) ethanol. The target concentrations of Cd and hormones were prepared by diluting them in Hutner’s medium. After 7 days of cultivation, the duckweed biomass was filtered using a vacuum pump (KNF Neuberger, Inc., Trenton, NJ, USA), weighed with a Precisa 180A balance (PAG Oerlikon AG, Zurich, Switzerland), and homogenized in liquid nitrogen using a mortar and pestle for further analysis.

### 4.2. Chemicals

All chemicals for Hutner’s medium, Bradford and Somogyi reagents, analysis of phytohormones, pigments, phytochelatins, and enzymes were bought from Merck KGaA (Darmstadt, Germany).

The standards of abscisic acid (ABA); auxins (AXs): indole-3-acetic acid (IAA) and phenylacetic acid (PAA), brassinosteroids (BRs): brassinolide (BL), 24-epibrassinolide (EBL), 28-homobrassinolide (HBL), 28-norbrassinolide (norBL), cathasterone (CT), castasterone (CS), 24-epicastasterone (ECS), typhasterol (TY) and 6-deoxotyphasterol (6dTY); cytokinins (CKs): *trans*-zeatin (tZ), *trans*-zeatin riboside (tZR), *trans*-zeatin-9-glucoside (tZ9G), *trans*-zeatin-7-glucoside (tZ7G), *trans*-zeatin riboside-*O*-glucoside (tZROG), *cis*-zeatin (cZ), *cis*-zeatin-9-glucoside (cZ9G), dihydrozeatin (DHZ), dihydrozeatin riboside (DHZR), dihydrozeatin-*O*-glucoside (DHZOG), *N*^6^-isopentenyladenine (iP), *N*^6^-isopentenyladenosine (iPR), *N*^6^-isopentenyladenosine-7-glucoside (iPR7G), *meta*-topolin (mT), *ortho*-topolin (oT), and gibberellic acid (GA_3_) were bought from OlChemIm (Olomouc, Czech Republic). The standard of melatonin (MT) was obtained from Merck KGaA (Darmstadt, Germany). The standards of pigments (chlorophyll *a*, chlorophyll *b*, α-carotene, β-carotene, astaxanthin, cryptoxanthin, neoxanthin, violaxanthin, zeaxanthin, and lutein) were bought from DHI (Horsholm, Denmark). The standards of PC_2_, PC_3_, PC_4_, and PC_5_ were bought from AnaSpec (Fremont, CA, USA).

### 4.3. Determination of Cadmium Content

For Cd determination, 1 g of fresh duckweed was suspended for 10 min in 20 mL of 100 µM Na_2_EDTA solution to remove Cd ions from the external surface. The biomass was dried in an oven for 12 h at 65 °C. The samples were suspended in 6 mL of 65% HNO_3_ (trace select purity) and heated for 15 min. The samples were analyzed by flame atomic absorption spectrometry (Solaar M6 spectrometer; TJA Solutions, Cambridge, UK) with a deuterium background correction system. The absorbance of Cd was measured using an air-acetylene flame with a spectral bandpass of 0.5 nm at 228.8 nm [95]. Additionally, in the 1st and 7th days of cultivation, 2 mL of Hutner’s medium was collected to measure the amount of Cd in the medium.

### 4.4. Determination of Photosynthetic Pigments

Approximately 0.5 g of plant powder was homogenized in 1 mL of 99.9% (*v*/*v*) MeOH using a ball mill (Omni Bead Ruptor Elite; OMNI International, Kennesaw, GA, USA) for 15 min. The obtained homogenate was then left at 4 °C for 12 h to isolate the pigments. Subsequently, the samples were centrifuged (2800× *g* for 10 min; MPW-55 Med. Instruments, Gliwice, Poland) and analyzed using the HPLC method [96].

### 4.5. Determination of Soluble Proteins and Monosaccharides

The content of soluble proteins and monosaccharides in *W. arrhiza* was measured spectrophotometrically using a Hitachi U-5100 UV-Vis spectrophotometer (Hitachi High-Tech Science Corporation, Tokyo, Japan). Bradford [97] method was employed for protein analysis, while Nelson [98] and Somogyi [99] methods, with modifications, were utilized for monosaccharides. The absorbance for proteins was measured at 595 nm, and for monosaccharides at 540 nm.

### 4.6. Determination of Malondialdehyde and H_2_O_2_ Content

Extracts of *W. arrhiza* were prepared to measure stress marker levels. Plant pellets (100 mg) were suspended in 2 mL of 0.1% (*w/v*) trichloroacetic acid (TCA), homogenized for 15 min, and centrifuged (2800× *g* for 10 min).

For MDA analysis, 0.5 mL of the supernatant was mixed with 2 mL of 0.5% thiobarbituric acid (TBA) in 20% TCA. This mixture was heated in a water bath at 95 °C for 20 min, cooled, and centrifuged (2800× *g* for 10 min). The MDA content was measured spectrophotometrically at 532 nm, with nonspecific absorption at 600 nm subtracted. The MDA-TBA complex concentration was calculated using a molar extinction coefficient of 155 mM/cm [100].

For H_2_O_2_ determination, 0.5 mL of supernatant was mixed with 0.5 mL of 10 mM potassium phosphate buffer (pH 7.0) and 1 mL of 1 M potassium iodide (KI) solution. A standard sample consisted of a 1 mM H_2_O_2_ solution with 10 mM potassium phosphate buffer (pH 7.0) and 1 M KI. After 60 min in darkness, the H_2_O_2_ levels were measured spectrophotometrically at 390 nm [101].

### 4.7. Determination of Glutathione and Phytochelatins

For sample preparation, 200 mg of plant pellet was suspended in 1 mL of trifluoroacetic acid containing 6.3 mM diethylenetriaminepentaacetic acid (DTPA), homogenized using a bead mill (50 Hz for 5 min), and centrifuged at 4 °C (2800× *g* for 10 min). The 250 µL of the supernatant was mixed with 450 µL of 200 mM 4-(2-hydroxyethyl)piperazine-1-propanesulfonic acid buffer containing 6.3 mM DTPA and 20 mM monobromobimane solution and then incubated at 45 °C for 30 min in the dark. The reaction was stopped by adding 300 µL of 1 M methanesulfonic acid solution. The endogenous levels of GSH and PCs were analyzed using the HPLC method [101,102].

### 4.8. Determination of Antioxidants

Previously, the enzymatic extracts were prepared to determine the antioxidant activity. Therefore, the duckweed samples were placed in the reaction mixture, which contained 50 mM phosphate buffer (pH 7.0), 1 mM EDTA, 1 mM phenylmethanesulfonylfluoride, 2% (*w*/*v*) polyvinylpyrrolidone, and 0.5% (*v*/*v*) Triton X-100. The mixtures were homogenized (15 min) and centrifuged (2800× *g*, 10 min, 4 °C).

For APX determination, 3 mL of the enzymatic extracts contained 0.1 mL of enzyme extract, 50 mM potassium phosphate buffer (pH 7.0), 1 mM H_2_O_2_, and 0.5 mM ascorbate. Absorbance was measured spectrophotometrically at the wavelength of 290 nm for 1 min of reaction (extinction coefficient 2.8 mM/cm) at 25 °C. One unit (U) of APX activity is defined as the amount of enzyme that oxidizes 1 µM of ascorbate per milligram of soluble protein per minute [103].

For CAT determination, 0.1 mL of the enzymatic extracts was suspended in 2.9 mL of 50 mM potassium phosphate buffer (pH 7.0) containing 1 mM H_2_O_2_. During the 1 min of reaction, the decrease in H_2_O_2_ absorbance was measured spectrophotometrically (240 nm, 25 °C). One U of CAT activity is defined as the amount of enzyme that decomposes 1 µM of H_2_O_2_ per milligram of soluble protein per minute [104].

For GR determination, 3 mL of the enzymatic extracts contained 0.1 mL of enzymatic extract, 50 mM potassium phosphate buffer (pH 7.6), 1 mM EDTA, 1 mM oxidized glutathione (GSSG), and 1 mM NADPH. The reaction started after the addition of NADPH at 25 °C. The absorbance was measured spectrophotometrically (340 nm, 1 min, extinction coefficient 6.2 mM/cm). One U of GR activity was determined from the NADPH oxidation rate by the decrease in absorbance [105].

For SOD determination, 3 mL of the enzymatic extracts contained 0.1 mL of enzymatic extract, 50 mM sodium carbonate (pH 10.2), 1 mM hydroxylamine, 0.1 mM EDTA, 0.024 mM solution of nitroblue tetrazolium, and 0.03% (*v*/*v*) Triton X-100. SOD activity was assayed spectrophotometrically at 560 nm. One U of SOD activity was assumed as the amount that causes a 50% inhibition of the photochemical reduction of NBT per mg of protein [106].

### 4.9. Analysis of Phytohormones

The quantitative analysis of phytohormones was performed using a Shimadzu LC-MS/MS-8050 system (Shimadzu Corporation, Kyoto, Japan).

For MT analysis, duckweed powder (0.5 g) was extracted in chloroform, homogenized, and centrifuged (2800× *g* for 10 min). After drying and suspension in 40% MeOH, the sample was analyzed according the procedure which was described in [74].

For BRs analysis, duckweed powder (200 mg) was extracted in 1 mL of 95% (*v*/*v*) MeOH, homogenized, and centrifuged (2800× *g* for 10 min). The supernatant (5 mL) was mixed (90 rpm, 12 h, 5 °C) and purified using solid-phase extraction (SPE) and analyzed according to Xin et al. [107].

For ABA, AXs, CKs, and GA_3_ analysis, duckweed powder (200 mg) was extracted in 50% (*v*/*v*) acetonitrile, homogenized, and centrifuged (2800× *g* for 10 min). The supernatant was purified using a Waters SPE Oasis HLB cartridge and eluted with 30% (*v*/*v*) acetonitrile. After drying and reconstitution in 50 µL of 30% (*v*/*v*) acetonitrile, the sample was analyzed according to Šimura et al. [108].

### 4.10. Statistical Analysis

Statistical analyses were conducted using Statistica 13.3 (TIBCO Software Inc., Palo Alto, CA, USA). Descriptive statistics were computed for the dataset, which was grouped by treatment, with each group consisting of five biological replicates. A one-way analysis of variance (ANOVA) was performed to assess statistical differences between groups, followed by Tukey’s Honestly Significant Difference test. The Shapiro-Wilk test was used to verify the assumption of normality required for ANOVA, with the significance level set at *p* < 0.05.

## 5. Conclusions

**Figure 8:**
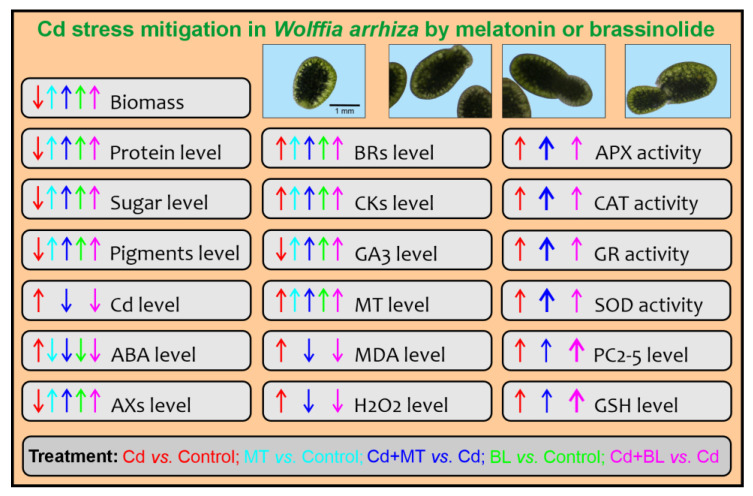
Summary of limiting the toxic effects of Cd on *W. arrhiza* by MT and BL. The application of MT or BL has demonstrated synergistic effects in reducing Cd toxicity by enhancing the plant’s tolerance to Cd and promoting better growth and development under stress conditions. The application of MT or BL offers a promising strategy to protect *W. arrhiza* from the toxic effects of Cd. Abbreviations are explained in the Abbreviations section.

In summary, exposure to Cd led to a dose-dependent decrease in both the growth and the content of metabolites in *W. arrhiza*. Conversely, Cd exposure enhanced the accumulation of PCs, stress markers, and increased antioxidant activity. The effect of Cd on the endogenous levels of phytohormones varied. The presented results confirm that both MT and BL limit the toxic effect of Cd (Figure 8); however, the addition of 25 µM MT was noticeably better than 0.1 µM BL in promoting growth and enhancing the content of protein, sugars, and most photosynthetic pigments. MT also reduced MDA and hydrogen peroxide (H_2_O_2_) levels while increasing antioxidant activity. In contrast, BL showed slightly greater efficacy than MT in enhancing the accumulation of GSH and PCs in the presence of Cd. Regarding phytohormone levels, MT was more pronounced in stimulating the biosynthesis of auxins, melatonin itself, and GA3, and in decreasing the level of ABA in *W. arrhiza* treated with Cd. Meanwhile, BL, to a greater extent than MT, stimulated the biosynthesis of BRs, free bases, and ribosides of CKs while decreasing CK glucosides in the presence of Cd ions. This variance in effective concentrations suggests differences in compound potency: MT requires higher concentrations to achieve strong effects, whereas BL is potent at much lower concentrations.

Our findings indicate that melatonin (MT) and brassinolide (BL) have significant potential as agents for enhancing plant resilience to heavy metal stress, such as cadmium exposure. The practical application of these hormones in phytoremediation systems, particularly in aquatic environments, holds promise as a sustainable strategy for environmental protection and agricultural management.

We plan to continue exploring the combined application of MT and BL to investigate potential synergistic effects. Future research will also include analyzing the impact of these hormones on plants subjected to other types of abiotic stress, such as salinity. Additionally, long-term experiments will be conducted to evaluate the durability of the observed effects.

## Figures and Tables

**Table 1:** Intracellular Cd concentration in *W. arrhiza* (mg/g dry weight) treated with Cd or/with MT or BL after 7 days of breeding, as well as the concentration of Cd in the medium (mg/L) on the 1st and 7th days of breeding. The results present the mean (n = 5, where n represents the number of biological replicates) ± standard deviation. Means with the same letters are not significantly different (*p* ≥ 0.05) according to Tukey’s post hoc test. Abbreviations are explained in the Abbreviations section.

Treatment	Cd Concentration in *W. arrhiza*	Cd Concentration in Medium (1 Day)	Cd Concentration in Medium (7 Day)
Control	0	0	0
0.1 µM Cd	0.026 ± 0.002 ^d^	0.007 ± 0.001 ^c^	0.005 ± 0.001 ^e^
1 µM Cd	0.31 ± 0.016 ^d^	0.101 ± 0.002 ^c^	0.088 ± 0.004 ^e^
10 µM Cd	4.088 ± 0.068 ^c^	1.435 ± 0.008 ^b^	1.155 ± 0.049 ^d^
100 µM Cd	42.076 ± 1.695 ^a^	11.652 ± 0.308 ^a^	10.299 ± 0.019 ^c^
0.1 µM Cd + 25 µM MT	0.021 ± 0.002 ^d^	0.007 ± 0.001 ^c^	0.007 ± 0.001 ^e^
1 µM Cd + 25 µM MT	0.28 ± 0.008 ^d^	0.103 ± 0.004 ^c^	0.094 ± 0.002 ^e^
10 µM Cd + 25 µM MT	3.232 ± 0.118 ^c^	1.447 ± 0.008 ^b^	1.225 ± 0.004 ^d^
100 µM Cd + 25 µM MT	38.342 ± 0.565 ^b^	11.556 ± 0.25 ^a^	10.906 ± 0.085 ^a^
0.1 µM Cd + 0.1 µM BL	0.024 ± 0.002 ^d^	0.007 ± 0.001 ^c^	0.006 ± 0.001 ^e^
1 µM Cd + 0.1 µM BL	0.284 ± 0.007 ^d^	0.103 ± 0.001 ^c^	0.091 ± 0.002 ^e^
10 µM Cd + 0.1 µM BL	3.495 ± 0.073 ^c^	1.442 ± 0.007 ^b^	1.191 ± 0.062 ^d^
100 µM Cd + 0.1 µM BL	39.403 ± 0.515 ^b^	11.538 ± 0.095 ^a^	10.785 ± 0.056 ^b^

**Table 2:** The endogenous level of photosynthetic pigments (µg/g fresh weight) in *W. arrhiza* treated with Cd or/with MT or BL after 7 days of breeding. The results present the mean (n = 5, where n represents the number of biological replicates) ± standard deviation. Means with the same letters are not significantly different (*p* ≥ 0.05) according to Tukey’s post hoc test. Abbreviations are explained in the Abbreviations section.

Treatment	Chlorophyll *a*	Chlorophyll *b*	α-Carotene	β-Carotene	Neoxanthin
Control	162.155 ± 2.69 ^e^	42.194 ± 2.2 ^e^	1.289 ± 0.01 ^de^	1.78 ± 0.07 ^c^	0.917 ± 0.04 ^e^
0.1 µM Cd	159.030 ± 1.13 ^e^	40.155 ± 0.73 ^e^	1.235 ± 0.01 ^ef^	1.701 ± 0.07 ^cd^	0.906 ± 0.02 ^e^
1 µM Cd	153.193 ± 1.15 ^e^	39.324 ± 0.56 ^ef^	1.095 ± 0.03 ^fg^	1.619 ± 0.03 ^cd^	0.864 ± 0.02 ^e^
10 µM Cd	140.7 ± 0.97 ^f^	33.991 ± 0.38 ^fg^	0.944 ± 0.02 ^gh^	1.411 ± 0.03 ^de^	0.739 ± 0.02 ^f^
100 µM Cd	65.017 ± 1.83 ^h^	18.041 ± 0.82 ^i^	0.591 ± 0.02 ^i^	0.895 ± 0.02 ^f^	0.473 ± 0.01 ^h^
25 µM MT	237.154 ± 1.76 ^a^	67.216 ± 1.26 ^a^	1.794 ± 0.02 ^bc^	2.911 ± 0.04 ^a^	1.442 ± 0.03 ^b^
0.1 µM Cd + 25 µM MT	228.022 ± 1.53 ^ab^	65.336 ± 0.95 ^a^	1.722 ± 0.07 ^c^	2.807 ± 0.03 ^ab^	1.389 ± 0.04 ^bc^
1 µM Cd + 25 µM MT	219.783 ± 0.92 ^bc^	63.692 ± 0.55 ^ab^	1.7 ± 0.01 ^c^	2.596 ± 0.06 ^ab^	1.286 ± 0.02 ^c^
10 µM Cd + 25 µM MT	160.743 ± 1.51 ^e^	48.853 ± 0.9 ^d^	1.415 ± 0.05 ^d^	1.705 ± 0.03 ^cd^	1.076 ± 0.04 ^d^
100 µM Cd + 25 µM MT	93.883 ± 2.69 ^g^	29.073 ± 1.0 ^gh^	0.791 ± 0.02 ^h^	1.155 ± 0.02 ^ef^	0.586 ± 0.02 ^g^
0.1 µM BL	222.803 ± 1.73 ^bc^	61.643 ± 1.23 ^ab^	2.399 ± 0.05 ^a^	2.793 ± 0.12 ^ab^	1.591 ± 0.04 ^a^
0.1 µM Cd + 0.1 µM BL	214.806 ± 7.35 ^cd^	58.608 ± 6.21 ^bc^	2.279 ± 0.13 ^a^	2.751 ± 0.28 ^ab^	1.453 ± 0.03 ^b^
1 µM Cd + 0.1 µM BL	205.117 ± 9.76 ^d^	54.036 ± 4.84 ^cd^	1.955 ± 0.17 ^b^	2.582 ± 0.31 ^b^	1.343 ± 0.03 ^bc^
10 µM Cd + 0.1 µM BL	158.028 ± 12.04 ^e^	42.560 ± 3.92 ^e^	1.673 ± 0.15 ^c^	1.635 ± 0.24 ^cd^	1.150 ± 0.16 ^d^
100 µM Cd + 0.1 µM BL	84.656 ± 5.31 ^g^	24.965 ± 3.34 ^h^	0.855 ± 0.08 ^h^	1.224 ± 0.19 ^e^	0.723 ± 0.02 ^f^
	**Violaxanthin**	**Astaxanthin**	**Zeaxanthin**	**Cryptoxanthin**	**Lutein**
Control	0.7 ± 0.01 ^f^	0.352 ± 0.02 ^e^	3.208 ± 0.06 ^d^	4.237 ± 0.08 ^d^	0.473 ± 0.01 ^e^
0.1 µM Cd	0.653 ± 0.03 ^fg^	0.328 ± 0.01 ^e^	3.143 ± 0.01 ^d^	4.141 ± 0.05 ^d^	0.422 ± 0.01 ^f^
1 µM Cd	0.625 ± 0.01 ^g^	0.317 ± 0.01 ^e^	3.079 ± 0.01 ^d^	4.061 ± 0.02 ^d^	0.392 ± 0.01 ^fg^
10 µM Cd	0.526 ± 0.01 ^h^	0.265 ± 0.01 ^f^	2.770 ± 0.02 ^e^	3.521 ± 0.02 ^e^	0.349 ± 0.02 ^h^
100 µM Cd	0.356 ± 0.02 l ^j^	0.147 ± 0.01 ^h^	1.853 ± 0.05 ^g^	2.634 ± 0.05 ^f^	0.242 ± 0.01 ^j^
25 µM MT	1.146 ± 0.04 ^a^	0.622 ± 0.03 ^a^	4.693 ± 0.03 ^a^	6.490 ± 0.03 ^a^	0.772 ± 0.01 ^a^
0.1 µM Cd + 25 µM MT	1.077 ± 0.03 ^b^	0.558 ± 0.02 ^bc^	4.577 ± 0.09 ^ab^	6.389 ± 0.07 ^a^	0.736 ± 0.03 ^ab^
1 µM Cd + 2 5 µM MT	0.966 ± 0.02 ^c^	0.472 ± 0.02 ^d^	4.442 ± 0.04 ^ab^	5.95 ± 0.07 ^bc^	0.605 ± 0.02 ^c^
10 µM Cd + 25 µM MT	0.702 ± 0.03 ^f^	0.346 ± 0.02 ^e^	3.764 ± 0.04 ^c^	4.325 ± 0.08 ^d^	0.405 ± 0.02 ^fg^
100 µM Cd + 25 µM MT	0.516 ± 0.01 ^h^	0.21 ± 0.02 ^g^	2.230 ± 0.03 ^f^	3.177 ± 0.03 ^e^	0.297 ± 0.02 ^i^
0.1 µM BL	0.961 ± 0.01 ^c^	0.582 ± 0.01 ^ab^	4.609 ± 0.09 ^ab^	6.253 ± 0.08 ^ab^	0.718 ± 0.01 ^b^
0.1 µM Cd + 0.1 µM BL	0.877 ± 0.03 ^d^	0.517 ± 0.03 ^c^	4.492 ± 0.26 ^ab^	6.116 ± 0.36 ^abc^	0.643 ± 0.03 ^c^
1 µM Cd + 0.1 µM BL	0.811 ± 0.04 ^e^	0.443 ± 0.02 ^d^	4.380 ± 0.2 ^b^	5.752 ± 0.26 ^c^	0.521 ± 0.03 ^d^
10 µM Cd + 0.1 µM BL	0.646 ± 0.03 ^fg^	0.313 ± 0.03 ^e^	3.568 ± 0.25 ^c^	3.965 ± 0.45 ^d^	0.376 ± 0.01 ^gh^
100 µM Cd + 0.1 µM BL	0.456 ± 0.02 ^i^	0.208 ± 0.02 ^g^	2.108 ± 0.11 ^fg^	3.291 ± 0.13 ^e^	0.276 ± 0.02 ^ij^

**Table 3:** The endogenous level of phytohormones (ng/g fresh weight) in *W. arrhiza* treated with Cd or/with MT or BL after 7 days of breeding. The results present the mean (n = 5, where n represents the number of biological replicates) ± standard deviation. Means with the same letters are not significantly different (*p* ≥ 0.05) according to Tukey’s post hoc test. Abbreviations are explained in the Abbreviations section.

	Control	0.1 µM Cd	1 µM Cd	10 µM Cd	100 µM Cd	25 µM MT	0.1 µM Cd + 25 µM MT	1 µM Cd + 25 µM MT	10 µM Cd + 25 µM MT	100 µM Cd + 25 µM MT	0.1 µM BL	0.1 µM Cd + 0.1 µM BL	1 µM Cd + 0.1 µM BL	10 µM Cd + 0.1 µM BL	100 µM Cd + 0.1 µM BL
PAA	4.07	3.899	3.63	2.939	2.762	5.787	5.509	4.992	4.2	3.814	6.328	6.157	5.91	4.301	3.477
	±0.35 ^de^	±0.2 ^def^	±0.19 ^ef^	±0.24 ^gh^	±0.19 ^h^	±0.38 ^ab^	±0.24 ^bc^	±0.26 ^c^	±0.25 ^d^	±0.2 ^def^	±0.21 ^a^	±0.26 ^a^	±0.22 ^ab^	±0.21 ^d^	±0.23 ^fg^
EBL	0.167	0.182	0.206	0.242	0.26	0.195	0.212	0.231	0.274	0.294	16.081	16.705	17.145	17.495	17.86
	±0.04 ^b^	±0.01 ^b^	±0.02 ^b^	±0.02 ^b^	±0.02 ^b^	±0.01 ^b^	±0.01 ^b^	±0.01 ^b^	±0.01 ^b^	±0.02 ^b^	±3.2 ^a^	±1.18 ^a^	±0.68 ^a^	±0.45 ^a^	±0.41 ^a^
HBL	0.633	0.77	0.841	1.132	1.318	0.635	0.775	0.839	1.228	1.322	1.720	2.016	2.195	2.633	3.014
	±0.15 ^f^	±0.05 ^f^	±0.02 ^f^	±0.06 ^e^	±0.05 ^e^	±0.12 ^f^	±0.04 ^f^	±0.04 ^f^	±0.18 ^e^	±0.07 ^e^	±0.3 ^d^	±0.09 ^c^	±0.08 ^c^	±0.04 ^b^	±0.1 ^a^
norBL	0.121	0.108	0.097	0.07	0.061	0.127	0.109	0.098	0.071	0.06	0.494	0.414	0.332	0.23	0.199
	±0.04 ^f^	±0.01 ^f^	±0.01 ^f^	±0.01 ^f^	±0.01 ^f^	±0.04 ^ef^	±0.01 ^f^	±0.01 ^f^	±0.01 ^f^	±0.01 ^f^	±0.11 ^a^	±0.01 ^b^	±0.01 ^c^	±0.01 ^d^	±0.01 ^de^
CT	1.956	3.292	4.346	4.171	3.41	2.778	3.597	4.879	4.567	3.861	3.47	3.703	5.148	4.788	4.198
	±0.61 ^h^	±0.14 ^fg^	±0.23 ^bcd^	±0.11 ^cde^	±0.16 ^fg^	±0.28 ^g^	±0.3 ^ef^	±0.38 ^ab^	±0.2 ^abc^	±0.32 ^def^	±0.56 ^f^	±0.17 ^def^	±0.24 ^a^	±0.16 ^abc^	±0.13 ^cde^
ECS	0.028	0.022	0.017	0.013	0.011	0.034	0.027	0.022	0.018	0.015	0.053	0.043	0.039	0.035	0.028
	±0.007 ^cd^	±0.003 ^de^	±0.002 ^ef^	±0.002 ^ef^	±0.002 ^f^	±0.005 ^bc^	±0.004 ^cd^	±0.003 ^de^	±0.003 ^ef^	±0.002 ^ef^	±0.007 ^a^	±0.003 ^b^	±0.006 ^b^	±0.002 ^bc^	±0.002 ^cd^
TY	0.184	0.184	0.205	0.278	0.21	0.24	0.244	0.251	0.323	0.242	0.571	0.597	0.62	0.675	0.589
	±0.05 ^e^	±0.01 ^e^	±0.01 ^de^	±0.01 ^cd^	±0.02 ^de^	±0.02 ^de^	±0.02 ^cde^	±0.02 ^cde^	±0.02 ^c^	±0.02 ^cde^	±0.12 ^b^	±0.01 ^ab^	±0.02 ^ab^	±0.03 ^a^	±0.02 ^b^
6 dTY	0.032	0.04	0.044	0.049	0.025	0.032	0.04	0.045	0.049	0.025	0.064	0.068	0.071	0.096	0.056
	±0.01 ^gh^	±0.01 ^efg^	±0.01 ^ef^	±0.01 ^de^	±0.01 ^h^	±0.01 ^fgh^	±0.01 ^efg^	±0.01 ^de^	±0.01 ^de^	±0.01 ^h^	±0.01 ^bc^	±0.01 ^bc^	±0.01 ^b^	±0.01 ^a^	±0.01 ^cd^
DHZ	0.386	0.417	0.415	0.312	0.253	0.394	0.426	0.462	0.379	0.323	0.452	0.481	0.488	0.396	0.35
	±0.03 ^ef^	±0.02 ^cde^	±0.03 ^cde^	±0.02 ^hi^	±0.02 ^i^	±0.02 ^def^	±0.01 ^bcde^	±0.02 ^abc^	±0.04 ^efg^	±0.01 ^gh^	±0.02 ^abcd^	±0.02 ^ab^	±0.05 ^a^	±0.03 ^def^	±0.03 ^fgh^
iP	0.408	0.41	0.458	0.356	0.075	0.467	0.488	0.597	0.484	0.124	0.63	0.645	0.718	0.596	0.162
	±0.02 ^de^	±0.01 ^de^	±0.03 ^cd^	±0.03 ^e^	±0.01 ^g^	±0.04 ^cd^	±0.04 ^c^	±0.03 ^b^	±0.05 ^c^	±0.03 ^fg^	±0.01 ^b^	±0.02 ^b^	±0.02 ^a^	±0.03 ^b^	±0.02 ^f^
iPR	0.351	0.372	0.452	0.791	0.539	0.361	0.369	0.456	0.761	0.536	0.496	0.512	0.65	1.186	0.701
	±0.02 ^g^	±0.03 ^g^	±0.02 ^f^	±0.02 ^b^	±0.03 ^e^	±0.01 ^g^	±0.02 ^g^	±0.01 ^f^	±0.02 ^bc^	±0.02 ^e^	±0.02 ^ef^	±0.03 ^ef^	±0.02 ^d^	±0.1 ^a^	±0.03 ^cd^
oT	0.23	0.441	0.458	0.657	0.751	0.215	0.428	0.454	0.631	0.711	0.25	0.482	0.502	0.68	0.827
	±0.01 ^g^	±0.02 ^f^	±0.02 ^ef^	±0.04 ^cd^	±0.03 ^b^	±0.01 ^g^	±0.02 ^f^	±0.02 ^ef^	±0.03 ^d^	±0.03 ^bc^	±0.02 ^g^	±0.02 ^ef^	±0.03 ^e^	±0.03 ^cd^	±0.03 ^a^
mT	0.015	0.022	0.054	0.079	0.111	0.019	0.022	0.053	0.073	0.013	0.016	0.028	0.064	0.085	0.152
	±0.001 ^e^	±0.003 ^e^	±0.01 ^d^	±0.01 ^c^	±0.03 ^b^	±0.003 ^e^	±0.004 ^e^	±0.01 ^d^	±0.01 ^cd^	±0.002 ^e^	±0.001 ^e^	±0.003 ^e^	±0.004 ^cd^	±0.01 ^c^	±0.01 ^a^

## Data Availability

Data is contained within the current article.

## Abbreviations

6dTY	6-deoxotyphasterol
ABA	abscisic acid
APX	ascorbate peroxidase
AX	auxin
BL	brassinolide
BR	brassinosteroid
CAT	catalase
Cd	cadmium
CK	cytokinin
CN	campestanol
CS	castasterone
CT	cathasterone
cZ	*cis*-zeatin
cZ9G	*cis*-zeatin-9-glucoside
DHZ	dihydrozeatin
DHZOG	dihydrozeatin-*O*-glucoside
DHZR	dihydrozeatin riboside
DTPA	diethylenetriaminepentaacetic acid
EBL	24-epibrassinolide
ECS	24-epicastasterone
GA_3_	gibberellic acid
GR	glutathione reductase
GSH	glutathione
HBL	28-homobrassinolide
IAA	indole-3-acetic acid
iP	*N*^6^-isopentenyladenine
iPR	*N*^6^-isopentenyladenosine
iPR7G	*N*^6^-isopentenyladenosine-7-glucoside
MDA	malondialdehyde
mT	*meta*-topolin
MT	melatonin
norBL	28-norbrassinolide
oT	*ortho*-topolin
PAA	phenylacetic acid
PC	phytochelatin
SOD	superoxide dismutase
TBA	thiobarbituric acid
TCA	trichloroacetic acid
TY	typhasterol
tZ	*trans*-zeatin
tZ7G	*trans*-zeatin-7-glucoside
tZ9G	*trans*-zeatin-9-glucoside
tZR	*trans*-zeatin riboside
tZROG	*trans*-zeatin riboside-*O*-glucoside
